# Vasorin at the crossroads: charting new paths in preeclampsia research

**DOI:** 10.3389/fcvm.2025.1703770

**Published:** 2025-11-12

**Authors:** Saravanakumar Murugesan, Lakshmi Saravanakumar, Jose Carlose Fernandez-Morales, Tamas Jilling, Dan E. Berkowitz

**Affiliations:** 1 Department of Anesthesiology and Perioperative Medicine, Division of Molecular and Translational Biomedicine, Heersink School of Medicine, University of Alabama at Birmingham, Birmingham, AL, United States; 2 Department of Pediatrics, Division of Neonatology, University of Alabama at Birmingham, Birmingham, AL, United States

**Keywords:** VASN, placenta, TGF-β, endothelial dysfunction, preeclampsia

## Abstract

Preeclampsia (PE) remains a leading cause of maternal and fetal morbidity and mortality worldwide, yet the precise molecular mechanisms driving its pathology are not fully understood. Recent research has established extracellular vesicles (EVs) as critical mediators in the development of PE-related vascular dysfunction. These nanosized, lipid-bound particles are secreted by cells and serve as a sophisticated system of intercellular communication, carrying functionally active cargo—including proteins, nucleic acids, and lipids—that can modulate physiological and pathological processes via autocrine, paracrine, and endocrine signaling. While the role of EVs-mediated communication in PE has been extensively studied, the specific functions of individual protein components within this cargo remain largely unexplored. Vasorin (VASN), a known regulator of the TGF-β signaling pathway, is a promising candidate for investigation in pregnancy disorder. This review synthesizes existing evidence on VASN's role in PE and discusses its potential contributions to disease pathogenesis. We will highlight the critical need for future research to elucidate VASN's function and evaluate its activity as a promising new therapeutic strategy for a spectrum of pregnancy-related disorders.

## Introduction

Despite decades of dedicated research, hypertensive pregnancy disorders, especially preeclampsia (PE), remain a major worldwide health concern (1). PE causes significant maternal and fetal morbidity and death, with the consequences differing by nation. Globally, about one in every ten pregnancies is impacted (2). In the United States, 9% of pregnant women develop hypertension, with 5% advancing to PE and 1.2% suffering severe symptoms or eclampsia. PE causes 8%–16% of the maternal mortality in high-income nations (1–3). Furthermore, women with a history of PE experience persistent vascular dysfunction after delivery, increasing their risk of cardiovascular disease (4). Despite existing evidence, several limitations prevent the incorporation of these findings into clinical treatment (5). Notably, they have a 40% increased risk of premature mortality. This also affects the fetus/neonate, with PE being the major cause of medically required premature birth and increasing the risk of severe morbidity associated with prematurity (1–5). The acute newborn care expenditures associated with these problems are expected to surpass a $1 billion annual burden in the United States (6).

Extracellular vesicles (EVs) are a class of lipid-bound nanoparticles, typically ranging from 20 to 130 nm in diameter, that are secreted from multivesicular bodies (MVB) (7). These vesicles encapsulate a diverse cargo of nucleic acids, proteins, and lipid rafts, which collectively serve to regulate physiological functions through autocrine and paracrine signaling pathways (8, 9). In pathological states, EVs are capable of mediating organ damage, and their specific cargo signatures have emerged as promising candidates for early diagnosis and disease prediction (5). While EV cargo proteins have recently been identified as key biomarkers in various disease conditions, their specific functions in normal placental development and the pathogenesis of PE remain incompletely defined. This review will focus on synthesizing the existing evidence for the function of the EVs cargo protein VASN in PE. It will highlight the critical need for future studies to fully explore the role of EVs protein signatures in pregnancy-related disorders.

### Pathophysiology of PE

The pathogenesis of PE remains a subject of intense investigation due to its complex and multifactorial etiology (10, 11). The development of PE is attributed to a combination of systemic and placental factors, including heightened systemic inflammation, immunological dysregulation, an imbalance of pro- and anti-angiogenic factors, and altered metabolic profiles (9, 10). Genetic predispositions, oxidative stress, and lipid metabolism are also thought to contribute to the disease's intricate pathophysiology (10, 11). Despite its varied origins, the central event in PE development is widely considered to be placental dysfunction. This is a two-stage process that begins with defective placentation in the first trimester. In early-onset PE (<34 weeks), this dysfunction is often linked to profoundly inadequate trophoblast invasion and failed remodeling of the maternal spiral arteries (12). This leads to severe placental ischemia. In late-onset PE (>34 weeks), the pathology is more associated with an imbalance in vascular and metabolic factors, resulting in reduced intervillous perfusion and chronic placental hypoxia. In both forms of the disease, the resulting placental stress and elevated shear forces on the syncytiotrophoblast (STB) layer led to widespread cellular injury (10, 11). This triggers the massive synthesis and release of bioactive mediators, including cellular debris, anti-angiogenic components, and EVs. These EVs, including exosomes, are critical carriers of pro-inflammatory cargo. Their systemic release into the maternal circulation is a key event in inducing the widespread endothelial dysfunction that defines the clinical symptoms of PE (13).

### EVs

EVs are a class of membrane-bound vesicles formed within the endosomal region of most eukaryotic cells. The multivesicular body (MVB) is a key endosome characterized by intraluminal vesicles that bud internally into the endosomal lumen (8, 14). When the MVB fuses with the cell surface, its contents are released as EVs. These vesicles encapsulate nucleic acids, proteins, lipids, non-coding RNAs, miRNAs, and other regulatory molecules, which are capable of exerting physiological changes through autocrine, paracrine, and endocrine signaling mechanisms (8, 14). The quantity and quality of placenta-derived EVs can provide valuable information about placental health. In a physiologically normal pregnancy, EVs are persistently shed from the syncytiotrophoblast (STB) into the maternal circulation. The release of these particles increases as the placenta develops and returns to non-pregnant levels within 48 h of delivery (14). EVs mediate cell-to-cell communication after their secretion. Numerous *in vitro* studies have shown the effect of placental vesicles on different target cells (14–16). Interestingly, EVs are capable of modulating the activity of neighboring cells, which is crucial for maintaining normal physiology during pregnancy (13–15). Nevertheless, pathological processes can alter the number and composition of these vesicles, potentially leading to the development of various pregnancy-related complications. In PE, placental remodeling causes morphological alterations and an expanded shedding of STB, which may contribute to endothelial dysfunction (14, 15). Notably, Chang et al. reported that plasma EVs from PE patients cause endothelial dysfunction by delivering soluble Fms-like tyrosine kinase-1 (sFlt-1) and soluble endoglin (sEng) to the endothelium (17). Furthermore, we recently reported that a decrease in EVs-VASN in the plasma of severe PE) patients mediates endothelial dysfunction (17).

### EVs cargo protein

EVs carry a diverse and dynamic protein cargo that reflects the physiological state of their cell of origin. These proteins, which are selectively packaged into EVs, can be broadly categorized into two main groups: those involved in EV biogenesis, structure, and trafficking, and those that actively mediate intercellular communication and biological function. Proteins such as TSG101 and Alix are key to the formation of multivesicular bodies (MVBs) and the subsequent release of EVs, providing a common structural signature for these vesicles. Other proteins on the EV surface, like integrins and tetraspanins (CD9, CD63, CD81), are crucial for targeting EVs to specific cells or organs, ensuring their cargo is delivered to the intended destination (9, 18). Once delivered, the functional proteins within the EV cargo can exert a wide range of biological effects on recipient cells, reprogramming their behavior to influence processes from immunity to tissue regeneration and disease pathogenesis. For instance, studies have shown that EVs from cancer cells can transfer proteins that promote metastasis and drug resistance (19), while EVs from stem cells can carry proteins that stimulate tissue repair in damaged organs (20). This sophisticated system of communication is also central to the pathophysiology of pregnancy-related disorders like PE. In this condition, EVs from the stressed placenta are released into the maternal circulation, carrying an altered protein cargo that induces systemic endothelial dysfunction, a hallmark of the disease (21).

### VASN

VASN or Slit-like 2 (Slitl2), was discovered in 2002 while studying a cDNA library obtained from mouse kidneys (22). Its initial name refers to its structural similarities to the Slit proteins, a group of guiding molecules involved in axon formation (23). The VASN gene in mice is found on chromosome 16 and spans approximately 11 kb, with two exons. This gene encodes a conventional single-pass type I transmembrane protein of 673 amino acids. This results in an estimated molecular weight of 72 kDa. This structure indicates that VASN has both an extracellular and a cytoplasmic domain, which may allow it to interact with both external signaling molecules and internal pathways. VASN protein found outside of cells, shares several structural features with the Slit protein family. These include a signal peptide (which helps guide the protein to its destination), leucine-rich repeats, and epidermal growth factor domains. VASN also contains a fibronectin type III domain. Following these domains is a hydrophobic transmembrane domain, which anchors the protein to the cell membrane, and a short cytoplasmic tail that extends into the cell (22, 23). Importantly, VASN is highly conserved across several species, with orthologs found in rats, zebrafish, chickens, and humans. Mouse VASN's amino acid sequence is more than 95% and 83% identical to its rat and human equivalents, indicating evolutionary conservation (24–27).

### VASN and the regulation of TGF-β signaling

Ikeda et al. (27) established a foundational understanding of VASN, demonstrating its expression in mature human tissues and elucidating its critical role in regulating TGF-β activity. TGF-β, an extracellular signaling molecule, influences diverse physiological and pathological processes, such as cell proliferation, differentiation, apoptosis, migration, and the development of diseases like cancer, cardiovascular problems, fibrosis, and skeletal disorders (26–30). Ikeda et al. notably demonstrated that reduced VASN expression in vascular smooth muscle cells following acute vascular injury contributes to the fibroproliferative response to vascular damage. This effect is mediated by VASN's extracellular domain, which binds to TGF-β family members and inhibits their signaling (27–29). This finding highlights VASN's crucial regulatory role in vascular health and disease. Moreover, Malapeira et al. (30) found that only the soluble form of VASN effectively inhibits TGF-β, and its production is tightly controlled by the metalloprotease ADAM17. Choksi et al. (31) discovered VASN as a target protein of HIF-1, naming it ATIA (anti-TNF*α* induced apoptosis). This study found that ATIA/VASN reduces ROS generation, protecting cells against TNF*α* and hypoxia-induced apoptosis. Interestingly, it was shown that VASN may be found not only in the cell membrane and extracellular space, but also in the mitochondria, where it exerts its anti-apoptotic action via altering thioredoxin-2 activity (31). Recent work by Taggi et al. (2023) notably provided the first evidence of VASN expression in human female reproductive tissues, specifically the ovary and endometrium (32).

VASN is known for its involvement in TGF-β regulation, but it has also been linked to cancer lately. Multiple studies have demonstrated VASN overexpression in various human malignancies, including hepatocellular carcinoma, breast cancer, and glioblastoma, where it significantly contributes to tumor development and angiogenesis. VASN has also been found as a possible biomarker linked to epithelial-mesenchymal transition (EMT) in thyroid and colorectal malignancies, which is a vital stage in cancer metastasis. In these situations, VASN appears to induce EMT via activating the YAP/TAZ and PI3 K/AKT signaling pathways (32, 33). While most current research focuses on VASN's pathobiology in disease, its physiological roles are remains incompletely understood. A significant exception is the 2018 work by Rimon-Dahari et al. (34), which looked at VASN's function in folliculogenesis in a mouse model system. This study found that VASN is produced by granulosa cells and increased by luteinizing hormone (LH), highlighting the importance of TGF-β in ovarian function (32). Conditional knockout (cKO) mice without VASN showed increased ovulation, hyperactive TGF-β signaling, and fewer atretic antral follicles (33). This study identified VASN as a novel regulator of murine folliculogenesis, suggesting a role in antral follicle survival and the establishment of the ovarian follicle pool. VASN's evolutionary conservation across mice and primates suggests that it may have comparable roles in the human ovary. Our study focuses on VASN's possible relevance in human reproduction, given its recognized roles as a TGF-β signaling inhibitor and anti-apoptotic factor that suppresses ROS generation.

### Role of TGF- β and ROS in pregnancy

TGF-β and ROS are critical for fertility and reproductive function. However, dysregulation can result in a variety of pregnancy related disorders (33, 35). TGF-β family members, including TGFβ1, TGFβ2, and TGFβ3, are abundant in mammalian reproductive organs. They have an impact on the development of the gonads and secondary sex organs, spermatogenesis, ovarian function, pregnancy immunoregulation, embryo implantation, and placental development (35). Physiological levels of Reactive Oxygen Species (ROS) serve as critical second messengers in a variety of reproductive processes, including follicular development, ovulation, sperm capacitation, and more. However, chronic and excessive ROS production leads to oxidative stress, contributing to several female reproductive dysfunction, including endometriosis, spontaneous abortion, preeclampsia, and preterm labor. TGF-β signaling is crucial for placental growth and function in PE, a primary cause of maternal and fetal morbidity and death. Dysregulation of this signaling is a defining feature of the illness. Furthermore, higher ROS levels lead to oxidative stress, which is a crucial factor in preeclampsia pathogenesis. Understanding the complex relationship between TGF-β and ROS is essential for creating effective solutions to manage pregnancy difficulties (33–37).

### Role of VASN in PE

The anti-angiogenic state characteristic of PE is primarily driven by an imbalance of soluble factors, notably elevated soluble fms-like tyrosine kinase-1 (sFlt-1) and decreased placental growth factor (PlGF) (38, 39). However, recent investigations have begun to explore novel upstream mediators of this widespread vascular dysfunction, with VASN emerging as a significant candidate. VASN, a type I transmembrane protein highly expressed in endothelial cells, is known primarily for its ability to inhibit TGF-β signaling by binding to and blocking TGF-β ligands, thereby regulating cellular proliferation and migration (40, 41). Several independent studies support the notion that VASN plays a protective role in maintaining vascular homeostasis, with a healthy placenta and endothelium maintaining stable levels of circulating VASN (33, 37, 41). The established hypothesis posits that in PE, placental stress leads to a reduction in these protective factors, including VASN. This deficiency is proposed to remove the brake on the TGF-β pathway (27, 37). Uncontrolled or hyper-activated TGF-β signaling within the endothelium is highly pro-inflammatory and pro-fibrotic, contributing to key PE features such as increased oxidative stress and subsequent systemic endothelial dysfunction (42, 43). This mechanism suggests that VASN deficiency acts as an upstream trigger, linking early placental-endothelial communication to the downstream hypertensive phenotype.

### VASN, EVs, and endothelial dysfunction

**Figure 1:**
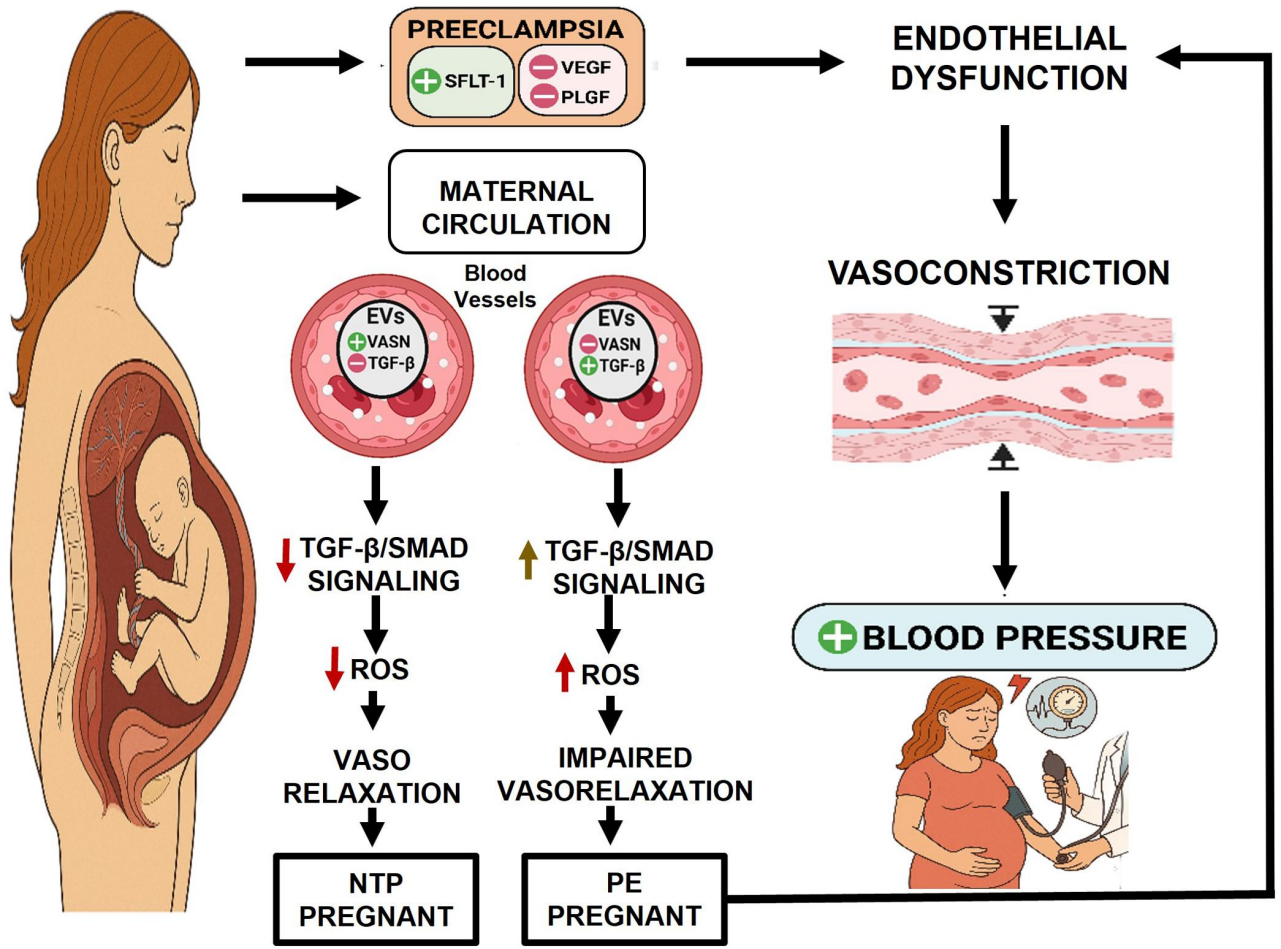
Proposed mechanistic model: VASN deficiency drives endothelial dysfunction and PE pathogenesis: NTP pregnant: during healthy gestation, the placenta releases EVs carrying sufficient VASN. VASN is hypothesized to maintain balanced physiological responses by modulating TGF-β signaling within endothelial cells. This results in homeostatic ROS levels and preserved endothelial function. PE pregnant: In PE, the placenta releases dysregulated EVs that are significantly deficient in VASN. The loss of VASN's modulatory control leads to uncontrolled or excessive TGF-β activation (e.g., hyperactivation of SMAD2/3 or non-SMAD pathways). This dysregulated signaling, combined with other VASN-deficiency effects, promotes increased ROS production. These combined moleculares events culminate in systemic endothelial dysfunction, which directly contributes to the clinical feature of maternal hypertension. Figure created with BioRender.com. sFLT-1, soluble fms-like tyrosine kinase-1; VEGF, vascular endothelial growth factor; PLGF, placental growth factor; EVs, Extracellular vesicles, VASN, vasorin, TGFβ, transforming growth factor beta; NTP, Normotensive pregnant patients and PE, preeclampsia. ROS, reactive oxygen species.

Our research aligns with and supports this broader mechanistic hypothesis by focusing on the role of VASN carried within EVs which mediate communication between the placenta and the maternal endothelium. We propose a compelling model: in normal pregnancy, VASN-containing EVs likely mediate adaptive cardiovascular responses, contributing to healthy physiological changes. Conversely, in PE, we hypothesize that dysregulated EVs, characterized by a deficiency in VASN and an altered cargo of other signaling molecules, actively contribute to the disease's pathobiology. This effect may involve promoting the dysregulation of TGF-β signaling, ultimately leading to endothelial and subsequent organ dysfunction (Figure 1). Supporting this model, we initially demonstrated that plasma EVs isolated from patients with severe PE (sPE) are functionally pathogenic, actively inducing endothelial dysfunction (37). To identify the specific molecular cargo responsible for this effect, a comprehensive proteomic analysis of urinary EVs was conducted (37). This unbiased approach confirmed a significantly altered proteome in sPE and, critically, identified VASN as a highly downregulated protein in sPE-EVs and placental tissue. Functional studies confirmed the significance of this finding: the pathogenic effects of sPE-derived EVs—such as impaired vasorelaxation and endothelial cell migration—were significantly reversed by VASN overexpression (37). This highlights VASN's critical potential role in maintaining vascular integrity. VASN, therefore, offers a window into the early loss of endothelial protective capacity, complementing the existing angiogenic markers which reflect the final vascular breakdown.

While the evidence strongly supports a role for EVs in PE pathogenesis, a critical assessment of the literature is essential to translate these findings. A major challenge in EVs research, particularly in maternal blood, is the variability in isolation methods and the difficulty in distinguishing placental EVs from those released by maternal tissues. This methodological challenge directly impacts the reproducibility of cargo measurements, including VASN. Furthermore, the observed decrease in VASN must be contextualized beyond PE specificity. It remains unclear whether reduced VASN levels are unique to severe PE or whether this change is a common feature shared with other hypertensive disorders of pregnancy, or simply a non-specific response to general systemic inflammation or underlying maternal comorbidities. Future studies must address these limitations by employing standardized isolation protocols and comparing VASN levels across a broad spectrum of hypertensive and inflammatory pregnancy states to establish VASN as a specific mechanistic driver of PE, rather than a general marker of vascular stress. Further research is also warranted to precisely map VASN's interaction with the sFlt-1/PlGF axis and to validate its utility as an early diagnostic or prognostic biomarker for this severe pregnancy complication (37, 44, 45).

### Clinical relevance and biomarker potential of EVs-VASN in PE

The established clinical utility of the sFlt-1/PlGF ratio highlights the critical need for biomarkers that accurately predict, diagnose, and stratify risk in PE. While VASN's potential is significant, any proposed biomarker must be rigorously contextualized against these validated assays, particularly concerning sensitivity, specificity, and the optimal gestational window for measurement. As summarized in Table 1, current clinical practice relies heavily on the angiogenic balance (sFlt-1/PlGF ratio) for short-term prediction and diagnosis from the mid-second trimester onward (44). In contrast, the potential role of EVs-VASN is not simply to replicate this ratio, but to provide a novel mechanistic indicator rooted in early endothelial and EVs dysregulation (37, 45). As VASN is a fundamental component of the pathological EVs cargo, its deficiency may precede or amplify the downstream angiogenic imbalance reflected by sFlt-1 and PlGF. Future clinical studies must focus on determining the gestational timing where VASN exhibits peak predictive value, comparing its performance (sensitivity and specificity) directly against the sFlt-1/PlGF ratio, and exploring its use in a multi-marker model. VASN is positioned to enhance our predictive algorithms by targeting a distinct, early-stage pathway, potentially identifying at-risk women before the severe angiogenic crisis fully manifests.

**Table 1:** Comparative clinical utility of established and emerging PE biomarkers (37–39).

Biomarker(s)	Utility (prediction/diagnosis)	Typical gestational timing	Performance (sensitivity/specificity)	Proposed role of VASN
sFlt-1/PlGF ratio	Rule-In/rule-out diagnosis, prognosis	Mid-Gestation (20wks) onward	High: NPV > 99% (Rule-Out PE within 1 week)	Mechanistic Indicator/Early Predictor
PlGF (placental growth factor)	Screening, short-term prediction	First and Second Trimesters (Screening)	Varies, but widely used in prediction algorithms	VASN deficiency may provide the upstream mechanism driving PlGF imbalance
PAPP-A (pregnancy-associated plasma protein A)	Risk stratification	First Trimester (11–14 weeks)	Low as a standalone marker; used in combination with UtA Doppler	Used in early risk assessment before VASN changes become significant
Uterine artery (UtA) doppler	Placental pathology (blood flow)	First and second trimesters	High when combined with biomarkers (75% DR for preterm PE).	Biophysical marker used alongside biochemical markers like VASN to enhance risk modeling
EVs-VASN	Mechanistic driver, early prediction	Primarily mid-to-late gestation	Requires large-scale validation	Hypothesized to be a novel mechanistic biomarker that reflects endothelial EVs dysregulation, offering an alternative pathway focus from the standard angiogenic profile.

## Conclusions and future directions

The compelling preclinical and translational findings presented in this paper establish a strong foundation for exploring EVs protein signatures, particularly VASN, as non-invasive biomarkers for PE. To translate this potential into clinical utility, future research must focus on several key areas. The initial priority is to validate the feasibility of using these VASN signatures as biomarkers in PE pathology. This will require the precise identification of their expression patterns across different stages of pregnancy and associated vascular pathology. By determining the concentration and specific protein content of these signatures throughout gestation, we can evaluate their potential as early biomarkers for PE and assess their ability to improve the predictive power of disease progression and clinical outcomes. Beyond their diagnostic potential, further studies are needed to determine the specific biological functions of these protein signatures. A critical area of investigation is to identify their preferred target cells and the cellular processes they modulate upon uptake. The placenta, as the vital interface between mother and fetus, is a key source of these protein signatures. Thus, exploring the cellular mechanisms by which placental cells respond to changes in the maternal vascular environment is essential. Overcoming these knowledge gaps will not only advance our understanding of pregnancy and its associated disorders but also open avenues for clinical intervention. We propose that the modulation of VASN activity represents a novel therapeutic strategy for a spectrum of pregnancy-related disorders, particularly in PE.
